# Relative effects of climate factors and malaria control interventions on changes of parasitaemia risk in Burkina Faso from 2014 to 2017/2018

**DOI:** 10.1186/s12879-024-08981-2

**Published:** 2024-02-07

**Authors:** Nafissatou Traoré, Taru Singhal, Ourohiré Millogo, Ali Sié, Jürg Utzinger, Penelope Vounatsou

**Affiliations:** 1 Swiss Tropical and Public Health Institute, Kreuzstrasse 2, CH-4123 Allschwil, Switzerland; 2 University of Basel, Petersplatz 1, CH-4001 Basel, Switzerland; 3 Nouna Health Research Centre, National Institute of Public Health, BP 02, Nouna, Burkina Faso; 4 Institut de Recherche en Sciences de la Santé/Centre National de Recherche Scientifique et Technologique, 01 BP, 2779 Bobo-Dioulasso, Burkina Faso

**Keywords:** Bayesian geostatistical modelling, Burkina Faso, Climatic factors, Interventions, Malaria Indicator Survey, Spatially varying coefficients

## Abstract

**Background:**

In Burkina Faso, the prevalence of malaria has decreased over the past two decades, following the scale-up of control interventions. The successful development of malaria parasites depends on several climatic factors. Intervention gains may be reversed by changes in climatic factors. In this study, we investigated the role of malaria control interventions and climatic factors in influencing changes in the risk of malaria parasitaemia.

**Methods:**

Bayesian logistic geostatistical models were fitted on Malaria Indicator Survey data from Burkina Faso obtained in 2014 and 2017/2018 to estimate the effects of malaria control interventions and climatic factors on the temporal changes of malaria parasite prevalence. Additionally, intervention effects were assessed at regional level, using a spatially varying coefficients model.

**Results:**

Temperature showed a statistically important negative association with the geographic distribution of parasitaemia prevalence in both surveys; however, the effects of insecticide-treated nets (ITNs) use was negative and statistically important only in 2017/2018. Overall, the estimated number of infected children under the age of 5 years decreased from 704,202 in 2014 to 290,189 in 2017/2018. The use of ITNs was related to the decline at national and regional level, but coverage with artemisinin-based combination therapy only at regional level.

**Conclusion:**

Interventions contributed more than climatic factors to the observed change of parasitaemia risk in Burkina Faso during the period of 2014 to 2017/2018. Intervention effects varied in space. Longer time series analyses are warranted to determine the differential effect of a changing climate on malaria parasitaemia risk.

**Supplementary Information:**

The online version contains supplementary material available at 10.1186/s12879-024-08981-2.

## Background

Malaria is a major global health problem affecting hundreds of millions of people every year and accounted for 619,000 deaths in 2021 [1]. Sub-Saharan Africa bears the largest burden contributing more than 80% of the total cases, mainly children under the age of 5 years [2]. Over the past two decades, an increasing number of partners and financial resources have enhanced malaria control efforts. The scale-up of interventions has saved millions of lives globally and cut malaria mortality by an estimated 36% from 2010 to 2020 [3]. Yet, malaria continues to cause severe morbidity and mortality, particularly in six sub-Saharan African countries, including Burkina Faso [2, 4]. Indeed, Burkina Faso contributed to 3.3% of global malaria cases in 2021 [1]. In 2020, 11,311,560 people were affected with a diagnostic confirmation rate of 92%, including 508,097 severe cases and 3,966 deaths [5]. As in many other malaria endemic countries, Burkina Faso mitigates the malaria burden through interventions such as insecticide-treated nets (ITNs), artemisinin-based combination therapies (ACTs), indoor residual spraying (IRS) and chemoprophylaxis. National ITN distribution campaigns and introduction of seasonal malaria chemoprevention (SMC) in children aged 36–59 months were carried out by the Ministry of Health since 2010 [6, 7]. However, malaria remained the main cause of outpatient attendance at health centres in all age groups in 2020 [6].

In Burkina Faso, malaria transmission is seasonal with a peak of incidence observed during and just after the rainy season [8]. This seasonal peak varies across the three major geographic zones linked with the duration of the rainy season; for instance, up to 3 months in the North, 6 months in the Centre, and 9 months in the South of the country [9]. Studies in different parts of the world have shown a relative impact of climatic factors on malaria, but the effect of climate variables varies according to regions. The development of malaria parasites in the mosquito depends on climatic and environmental factors [10]. As regards climatic factors, temperature and humidity are particularly relevant [10]. Environmental changes may result in conditions that favour the spread of malaria, offsetting the impact of preventive measures, such as ITNs, IRS, ACT and SMC, thus putting more people at risk of malaria [11]. Altitude and rainfall have been significantly associated with malaria cases in south Sumatra, Indonesia [12]. Beloconi et al. (2023), using transmission models, have reported that temperature had a significant negative effect on malaria incidence in the lowland of Kenya [13]. Another study in West Africa, using a Bayesian modelling approach, has shown that the average annual temperature had negative association with malaria incidence [14]. It has also been demonstrated that, besides the important effects of interventions, living in urban areas was associated with a lower odds of malaria, while rainfall, land surface temperature (LST) day and night were associated with a higher odds in Uganda [15]. Studies conducted in Kenya and Uganda found that LST and rainfall showed a negative and positive association with malaria, respectively [16, 17]. Using Bayesian geostatistical modelling, previous studies have identified several environmental factors that were significantly associated with malaria transmission in Burkina Faso. For example, Samadoulougou et al. (2014), have reported that rainfall and maximum temperature were respectively positively and negatively associated with malaria after accounting for spatial dependence [18]. Likewise, Diboulo et al. (2016) and Rouamba et al. (2019) found that night LST and rainfall, respectively, showed a negative and positive association with malaria [19, 20].

While there is a growing body of literature studying the impact of climatic factors on malaria using spatial and temporal models at the individual level, the relationship between changes in climate and interventions on malaria is not well established. In this study, we estimated the differential effect of malaria interventions and climatic factors on the temporal changes of malaria parasite prevalence of children under the age of 5 years. The analysis is based on data from two Malaria Indicator Surveys (MIS) conducted in 2014 and 2017/2018, employing Bayesian geostatistical modelling. The effects of malaria interventions are estimated at country and regional levels, readily adjusted for climatic confounders. Furthermore, we predicted the geographical distribution of parasitaemia risk at high spatial resolution in 2014 and 2017/2018 and the posterior probability of reduction in parasitaemia risk between the two surveys and of the number of infected children.

## Methods

### Country profile

**Fig. 1:**
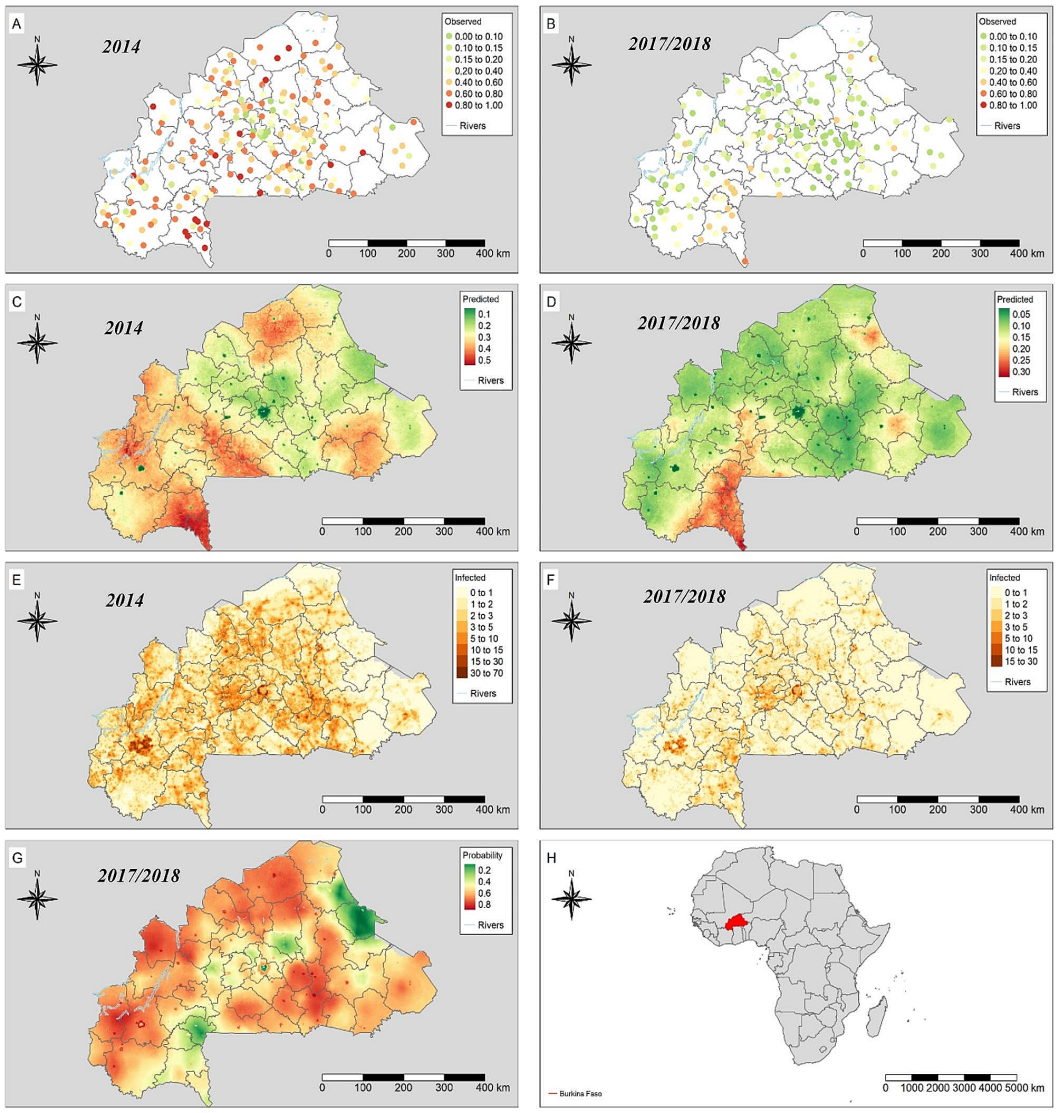
Observed malaria prevalence and survey locations of MIS 2014 (**A**) and 2017/2018 (**B**); predicted parasitaemia risk in 2014 (**C**) and 2017/2018 (**D**); distribution of estimated number of infected children per km^2^ pixel in 2014 (**E**) and 2017/2018 (**F**); posterior probability of reduction in parasitaemia risk (**G**); location diagram of Burkina Faso (**H**)

Burkina Faso is a landlocked country in West Africa, which belongs to the Sahel region. It is mainly located on a savannah plateau. On average, Burkina Faso receives between 500 mm and 1,000 mm of rainfall mainly between June and September and has an average annual temperature of around 28 °C [21]. The country had a population slightly more than 20 million in 2020, all of whom are at risk of malaria [9]. Malaria was responsible for 60% of the hospitalisations and was the largest contributor to mortality for children under the age of 5 years in 2021 [9, 22]. *Plasmodium falciparum* is the parasite responsible for 90% of malaria cases and *Anopheles gambiae* is the most efficient malaria vector species in Burkina Faso [23]. The LST day and LST night have increased in Burkina Faso over the past decade (see Fig. 1B in additional file 1). In recent years, precipitation increased from May to the end of August, while the temperature (day and night) decreased. However, from September to November, precipitation decreased and temperature increased in the recent past (see Fig. 1B in additional file 1).

### Survey data

The National Institute for Statistics and Demography (INSD) of Burkina Faso conducted a first MIS from October to November 2014 and a second MIS from November 2017 to March 2018. Hence, the MIS 2014 was carried out shortly after the end of the rainy season, while the MIS 2017/2018 in the dry season. For each survey, a random sample of 252 clusters (200 in rural and 52 in urban settings) with 6,552 households were selected through a stratified two-stage sampling procedure. The clusters were the census units established by INSD in 2006 [24]. Each region is divided into urban and rural areas to form the two sampling clusters within the region and the samples were drawn independently in each cluster. At the first stage, 252 clusters were drawn with a probability proportional to the size of households in the cluster during the mapping operation for the 2006 census. At the second sampling stage, a count of households in each of these 252 clusters provided a list of households from which 26 households were selected with equal probability systematic sampling. As part of the final sampling, one in two households were randomly selected and all children under the age of 5 years tested for parasitaemia. Two malaria diagnostic tests were performed; (i) a rapid diagnostic test (RDT) and (ii) a blood film examined under a microscope [25, 26]. Our data analysis included 224 clusters for 2014 and 194 clusters for 2017/2018 that had geo-referenced information available. The number of households used was 6,449 in 2014 and 6,318 in 2017/2018. The surveys included information about interventions and ACT at the cluster locations. We created intervention coverage indicators, namely the proportion of children and adults using ITNs, the proportion of household owning ITNs and the proportion of children under the age of 5 years with fever 2 weeks prior to the survey treated by ACT.

### Climatic data

Climatic data were downloaded from remote sensing sources for the full year prior to the end of the surveys, i.e. from November 2013 to November 2014 for MIS 2014 and from March 2017 to March 2018 for MIS 2017/2018. Day and night LST were extracted from Moderate Resolution Imaging Spectroradiometer (MODIS) at a spatial resolution of 1 × 1 km^2^ and a temporal resolution of 8 days. Night light, distance to permanent water and crops coverage were obtained from MODIS at 1 × 1 km^2^ spatial resolution and a temporal resolution of 12 months. Dekadal rainfall data were obtained from the US Early Warning and Environmental Monitoring System (EWES) at 8 × 8 km^2^ spatial resolution and a temporal resolution of 5 days. The climatic data were extracted at their original spatial and temporal resolution and summarised by their average for the year prior to the end of the surveys. They were matched spatially to the MIS locations and to the locations of the predictions. Altitude was obtained from the Shuttle Radar Topographic Mission (SRTM) using the digital elevation model. The high spatial resolution population data were downloaded from WorldPop at 1 × 1 km^2^ spatial resolution. Table 1 describes the sources and the spatio-temporal resolution of the data used.

**Table 1:** Description of the sources, spatio-temporal resolution of the climatic data for the period of 2014 to 2017 of Burkina Faso

Data	Source	Period	Spatial resolution	Temporal resolution
Rainfall	EWES	2014–2017	8 × 8 km^2^	5 days
Day-time land surface temperature	MODIS	2014–2017	1 × 1 km^2^	8 days
Night-time land surface temperature	MODIS	2014–2017	1 × 1 km^2^	8 days
Night light	MODIS	2014–2017	1 × 1 km^2^	1 year
Crop coverage	MODIS	2014–2017	1 × 1 km^2^	1 year
Distance to permanent water body	MODIS	2014–2017	1 × 1 km^2^	1 year
Altitude	SRTM	2014–2017	NA	NA
Population data	WorldPop	2014–2017	1 × 1 km^2^	NA

EWES, Early Warning and Environmental Monitoring System; MODIS, Moderate Resolution Imaging Spectroradiometer; NA, not applicable; STRM, Shuttle Radar Topographic Mission; WorldPop, World population

### Statistical analysis

Descriptive statistics were employed to examine the characteristics of the data and for summarising the variables. Prior to fitting the Bayesian model, collinearity between all pairs of predictors was assessed using non-spatial regression methods based on values of variance inflation factor (VIF); predictors with VIF > 5 were excluded from further analysis.

Bayesian geostatistical bivariate logistic regression was used first to assess the association between all predictors and parasitaemia risk. We assumed that the number of children under the age of 5 years, tested positive by blood microscopy, follows a binomial distribution. A multivariate model was developed to predict the prevalence at the two survey years using only the important climatic factors and interventions, as predictors that were identified by the bivariate analysis. We standardised the predictors by subtracting their mean and dividing the difference by their standard deviation (SD) to facilitate comparison of their effects at the country level. The spatial correlation was taken into account by introducing location random effects. They were modelled by a multivariate normal distribution with an exponential function of distance between any pair of locations. Furthermore, the model allowed us to estimate the minimum distance at which the spatial correlation is lower than 5% (range parameter). Bayesian kriging was carried out to predict parasitaemia risk over a 2 × 2 km^2^ resolution grid. The posterior probability of a decline in parasitaemia risk between the two survey years was determined using samples from the corresponding posterior predictive distributions (PPD). We draw a sample of size 100 from each PPD at grid cell level and computed their differences (i.e. predicted parasitaemia risk in 2017/2018 minus parasitaemia risk in 2014). The proportion of samples with a positive difference at each grid cell estimates the posterior probability of a decline in parasitaemia risk.

We assessed the effects of the changes in the coverage of interventions and climatic factors on changes in parasitaemia prevalence between the two surveys by fitting a Bayesian geostatistical model to the malaria survey data of 2017/2018. The changes in interventions (i.e. the differences in 2017/2018 coverage compared to the 2014 coverage) and the changes in climatic factors (i.e. differences in 2017/2018 values from those in 2014) were included as covariates in the logit of parasitaemia risk. The logit of parasitaemia in 2014 was considered as an offset term to enable changes in the risk between the survey years to be modelled on the logit scale. The geographical misalignment of the locations between the two surveys was taken into account by predicting the prevalence of the first survey at the locations of the second survey.

The number of children infected with *Plasmodium* in each survey year was estimated by multiplying population data obtained from WorldPop [27] at 1 km^2^ spatial resolution with the predicted pixel-level malaria parasitaemia prevalence. The number of children under the age of 5 years in a specific pixel was estimated by multiplying the population counts by 0.183 [28] and 0.180 [29], which are the proportions of population under the age of 5 years in 2014 and 2017/2018, respectively. Regional and national estimates of the number of infected children under 5 years old were obtained by aggregating the pixel-level estimates within each region and across the country. The relative reduction of the number of infected children was calculated by dividing the difference of the numbers of infected of that age group between the two surveys by the number of infected in the first survey. The estimates of the total number of infected children under 5 years old, both by region and for the entire country, were divided by the total population of that age group. This allowed us to obtain population-adjusted prevalence estimates at both regional and national levels, respectively.

A spatially varying coefficients model was fitted to estimate the effects of malaria interventions at a regional level. A conditional autoregressive (CAR) prior distribution was considered to introduce a neighbour-based spatial structure on the regression coefficients related to each intervention effect in the study. Neighbours were defined as the adjacent areas regions and used to create a matrix of spatial weights taking the value of one for a direct neighbour and zero otherwise [19, 30]. We produced maps of the random effects of the interventions. The random effect was estimated as the difference between the regional effect and the national effect. Following this approach, a negative value indicates that the effect of intervention at regional level is statistically lower than the national average.

Geostatistical models were fitted in Integrated Nested Laplace Approximation (INLA) [31] package in R (version 4.1.2) [32]. The spatially varying coefficients model was implemented in OpenBugs (version 3.2.3, Imperial College and Medical Research Council; London, UK) [33]. We summarised the parameter estimates by computing the posterior medians of odds ratio (OR) and their corresponding 95% Bayesian credible intervals (BCIs). The effect of a covariate was considered statistically important when the 95% BCI did not include one. A detailed description of the analysis is provided in supplementary file (see Additional file 2).

## Results

### Descriptive analysis

A description of the survey data and climatic factors is given in Table 2. Maps of the observed prevalence for both surveys are shown in Fig. 1. The number of screened children under the age of 5 years were 5,082 and 4,417 in the 2014 and 2017/2018 MIS, respectively. The prevalence of malaria was reduced from 44.7% in 2014 to 16.2% in 2017/2018. The lowest prevalence was observed in the Centre (9.3% in 2014 and 6.6% in 2017/2018) and the highest in the region of South-West (60.2% in 2014 and 39.0% in 2017/2018) (see Fig. 1A in additional file 1, and Table 3A in additional file 3). Among the regions, the highest decrease in the prevalence of malaria from 2014 to 2017/2018 occurred in the region of North with a relative reduction of 75.7%. The proportion of the population that slept under an ITN the night before the survey decreased by 33.7% comparing 2014 with 2017/2018. However, the proportion of fever episodes treated with ACT increased by 65.2%.

**Table 2:** Survey information, climatic factors and malaria intervention coverage in 2014 and 2017/2018 in Burkina Faso

Variable	MIS 2014 (n, %, 95% CI)	MIS 2017 /2018 (n, %, 95% CI)
Number of clusters	224	194
Number of households	6,449	6,318
Number of children under the age of 5 years	5,082	4,417
Parasitaemia prevalence	44.7%	16.2%
**Malaria interventions**		
**ITN ownership**		
Households with at least one ITN	89.9% (88.7–91.0%)	75.1% (73.3–77.0%)
Households with at least one ITN for every two people	47.7% (46.0-49.3%)	31.7% (30.0-33.4%)
Population with access to an ITN in their household	75.4% (74.1–76.7%)	58.4% (56.6–60.1%)
**ITN use**		
Population that slept under an ITN the previous night	67.4% (66.0–69.0%)	44.7% (43.0-46.5%)
Children under 5 years old who slept under an ITN	75.5% (73.8–77.3%)	55.1% (52.7–57.5%)
Proportion of existing ITNs used the previous night	86.1% (85.0-87.3%)	77.4% (75.6–79.0%)
**Case management**		
Children with fever treated with ACT	15.2% (13.0-17.3%)	43.6% (39.5–47.6%)
Household sprayed in the past months	0.5% (0.2–0.8%)	-
Households with at least one ITN and/or IRS in the past 12 months	89.7% (88.5–91.0%)	75.0% (73.0–77.0%)
**Climatic factors**		
LST night^1^	21.3 (21.2–21.4)	22.0 (21.7–22.0)
LST day^1^	35.5 (35.3–35.7)	35.9 (35.7–36.2)
Rainfall^1^	72.6 (70.4–74.8)	63.77 (62.6–65.0)
Night light^1^	6.59 (4.4–8.7)	7.00 (4.8–9.1)
Altitude^1^	309 0.0 (303.9–314.0)	309.5 (304.0-315.0)
Distance to permanent water^1^	46.7 (45.7–47.7)	118.0 (110.0-126.5)
Crop coverage^1^	0.44 (0.34–0.54)	0.43 (0.23–0.62)

^1^annual average, Abbreviations: ITN, insecticide-treated net, ACT, artemisinin-based combination therapy; LST, land surface temperature; MIS: Malaria Indicator Survey

### Effect of interventions and climatic factors on the geographical distribution of parasitaemia risk from 2014 to 2017/2018

Results from the geostatistical bivariate model (Table 3) showed that the relationship between the intervention coverage indicators and the malaria prevalence was not statistically important in 2014, except of the proportion of households with at least one ITN for every two people. However, there is evidence that in 2017/2018, ITN ownership, ITN use and ACTs were associated with a decrease of parasitaemia risk. Night LST, night light and distance to permanent water were negatively associated with parasitaemia risk in both periods. Altitude was statistically important in 2014 but not in 2017/2018.

The final multivariate geostatistical models showed that more climatic factors were associated with the geographical distribution of parasitaemia risk in 2014 than in 2017/2018 (Table 4). In 2014, none of the intervention indicators included in the analysis was statistically important. In 2017/2018, only the proportion of children who slept under an ITN was important with an OR of 0.82 (95% BCI: 0.70–0.96). The spatial range parameter was smaller, while the spatial variance was higher in 2014 compared to 2017/2018. The LST night was statistically important for both time points with an OR of 0.72 (95% BCI: 0.64–0.93) in 2014 and 0.89 (95% BCI: 0.81–0.96) in 2017/2018. There were no statistically important interactions between malaria interventions and LST night (see Table 3B in additional file 3). Night light was also negatively associated to parasitaemia risk in 2014 (OR = 0.47, 95% BCI: 0.38–0.59) and 2017/2018 (OR = 0.49, 95% BCI: 0.37–0.64). An altitude of above 307 m was associated with a 28% lower odds of parasitaemia in 2014. Figure 1C and D depict the predicted prevalence in 2014 and 2017/2018 over a 2 × 2 km^2^ resolution grid. In 2014, we observed a high-predicted parasitaemia risk (above or equal to 50%) in some areas, such as South-West, Centre-West and Hauts-Bassins. The posterior probability of reduction in parasitaemia risk was more than 0.50 in most part of the country. The highest decline in malaria prevalence was reported in the regions of Centre-East, Plateau-Central and Centre, while the least decline occurred in the South-West region (Fig. 1G).The number of infected children decreased from 704,202 in 2014 to 290,189 in 2017/2018 (Table 5), corresponding to a relative reduction in the country of 58.8%. The number of infected children was reduced in all regions (Fig. 1E and F). Reduction rates above the national average were observed in the regions of Centre-East, Hauts-Bassins, Boucle du Mouhoun, North and Centre-Southern, whereas the lowest was in the South-West (38.3%) (Table 5). In both surveys, the highest and lowest estimated numbers of children infected were observed in the regions of Hauts-Bassins and Centre, respectively. In addition, a reduction of population-adjusted prevalence of 63.1% was observed overall.

### Effects of changes in intervention coverage and climatic factors on the decline in parasitaemia prevalence from 2014 to 2017/2018

**Fig. 2:**
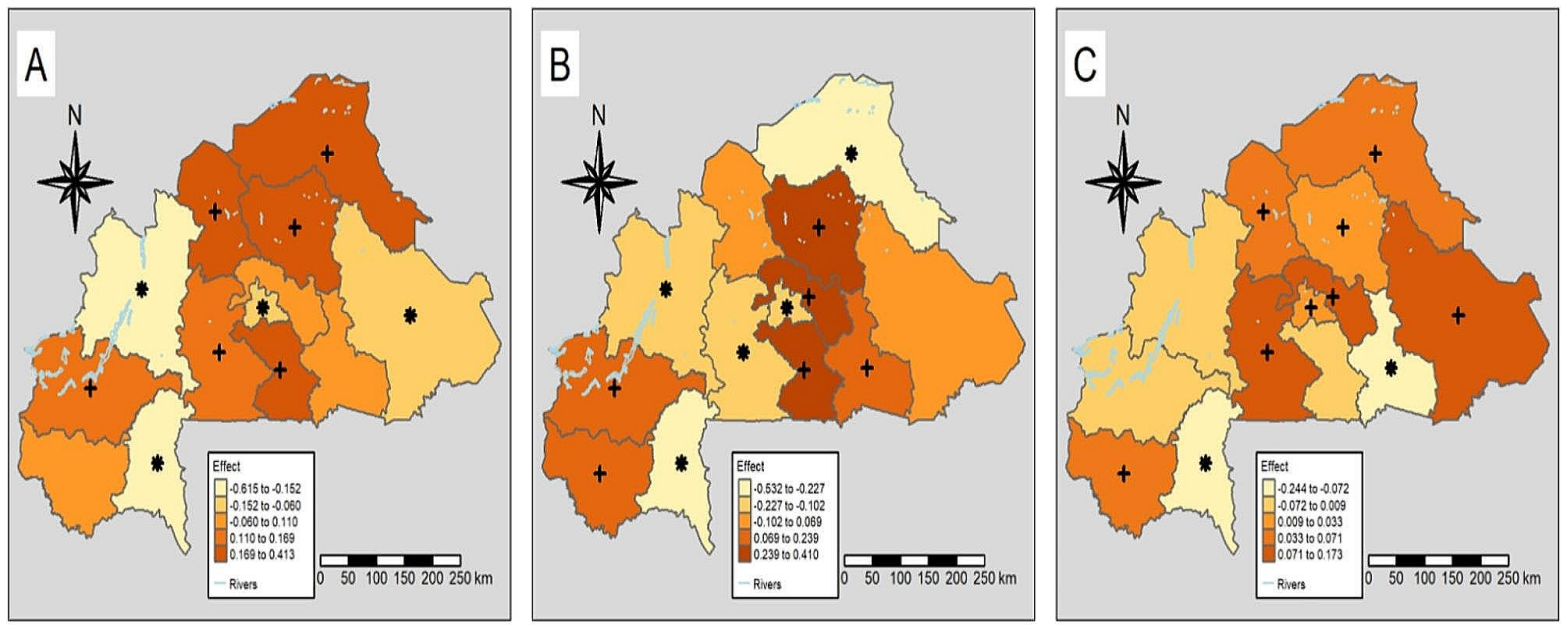
Effect of proportion of households with at least one ITN for every two people at regional level (**A**); effect of proportion of children who slept under an ITN at regional level (**B**) and effect of ACT coverage at regional level (**C**) *Statistically important effect less than national effect +Statistically important effect higher than national effect

The effects of changes in intervention coverage indicators and climatic factors between the two surveys on the parasitaemia risk decline are shown in Table 6. The results suggest that a 1% increase in the proportion of children who slept under an ITN was associated with a decrease of parasitaemia odds by 24%. The change in ACT coverage and in the proportion of households with at least one ITN for every two persons showed no statistically important effects in the parasitaemia risk decline. A one-unit increase in the change in night light was associated with a 15% lower odds of parasitaemia between the two surveys. Changes in LST night and distance to permanent water between 2014 and 2017/2018 were not associated with a decrease of parasitaemia risk. The residual spatial variation in the changes of parasitaemia risk accounted for 71% of total variation. The geographical distribution of the effects of interventions at regional level (Fig. 2) indicates that ITN ownership had a statistically important effect lower than the national effect in the regions of South-West, Boucle du Mouhoun, Centre and East. In North, Sahel, Centre-North, Centre-West, Centre-South and Hauts-Bassins, this effect was stronger than the national average. The effect of the proportion of children who slept under an ITN was statistically different from the national average in all regions, except for the regions of North and East. ACT was statistically important and more effective in the regions of Hauts-Bassins, Boucle du Mouhoun and Centre-South. ACT was less effective than nationally in the regions of South-West and Centre-East.

**Table 3:** Posterior odds ratios (ORs) and 95% Bayesian credible intervals (BCIs) estimated by bivariate geostatistical logistic regression models, fitted to MIS 2014 and 2017/2018 data from Burkina Faso

Predictor	MIS 2014 OR (95% BCI)	MIS 2017/2018 OR (95% BCI)
Proportion of households with at least one ITN for every two people (ITN ownership)	0.72 (0.60–0.85)^a^	0.85 (0.72–0.94)^a^
Proportion of children who slept under an ITN (ITN use)	1.06 (0.85–1.32)	0.86 (0.70–0.94)^a^
ACTs	0.87 (0.75–1.01)	0.84( 0.72–0.98)^a^
Proportion of households with at least one ITN and/or IRS	1.31 (0.93–1.86)	0.72 (0.16–1.30)
LST day	0.73 (0.59–1.12)	0.76 (0.63–1.09)
LST night	0.58 (0.48–0.69)^a^	0.83 (0.70–0.97)^a^
Rainfall	1.02 (0.85–1.21)	1.03 (0.80–1.30)
Night lights	0.43 (0.35–0.53)^a^	0.52 (0.41–0.66)^a^
Altitude*		0.98 (0.82–1.16)
[201–307 m] [307–545 m]	1.00 0.72 (0.51–0.96)^a^	1.00 0.97 (0.82–1.16)
Crop coverage	0.69 (0.58–1.20)	0.89 (0.72–1.11)
Distance to permanent water	0.87 (0.75–0.99)^a^	0.79 (0.63–0.97)^a^

^a^ Statistically important effect, ^*^ The cutoffs are based on the tertiles of the distribution of altitude at the surveys locations, ITN, insecticide-treated net; ACT: artemisinin-based combination therapy, LST: land surface temperature

**Table 4:** Posterior odds ratios (ORs) and 95% Bayesian credible intervals (BCIs) estimated by multivariate geostatistical logistic regression models fitted to MIS 2014 and 2017/2018 data from Burkina Faso

Predictor	MIS 2014 OR (95% BCI)	MIS 2017/2018 OR (95% BCI)
LST night	0.72 (0.64–0.93)^a^	0.89 (0.81–0.96)^a^
Night light	0.47 (0.38–0.59)^a^	0.49 (0.37–0.64)^a^
Distance to permanent water body	0.83 (0.72–0.94)^a^	0.71 (0.57–0.85)^a^
Altitude^*^		
[201–307 m] [307–545 m]	1.00 0.72 (0.51–0.96)^a^	-
Proportion of households with at least one ITN for every two people (ITN ownership)	1.06 (0.24–1.48)	1.04 (0.86–1.20)
Proportion of children who slept under ITN (ITN use)	-	0.82 (0.70–0.96)^a^
ACTs	-	1.22 (0.93–1.40)
**Spatial parameters**		
Spatial variance	0.41 (0.19–0.71)	0.32 (0.15–0.61)
Non spatial variance	0.31 (0.16–0.49)	0.29 (0.14–0.48)
Range (km)	129.4 (57.1-367.5)	303.7 (189.2-371.1)

^a^ Statistically important effect, ^*^ The cutoff is based on the tertiles of the distribution of altitude at the surveys locations, range is minimum distance at which the spatial correlation in no longer or less than 5%, ITN: insecticide-treated net, ACT: artemisinin-based combination therapy, LST: land surface temperature

**Table 5:** Estimated number of children infected with *Plasmodium* and population adjusted prevalence in 2014 and 2017/2018 in Burkina Faso

Region	No. of infected children in 2014	No. of infected children in 2017/2018	Relative reduction of infected children (%)	Population adjusted prevalence in 2014 % (95% BCI)	Population adjusted prevalence in 2017/2018 % (95% BCI)	Relative population adjusted prevalence reduction (%)
Boucle Du Mouhoun	98,740	35,930	63.6	31.7 (31.5–32.1)	10.6 (10.5–11.1)	66.4
Centre	18,942	9,058	52.2	3.61 (3.3–3.9)	1.4 (1.4–1.6)	60.9
Cascades	43,244	19,489	54.9	29.5 (29.4–29.8)	11.1 (11.0-11.5)	62.4
Centre-East	65,144	22,284	65.8	25.1 (25.01–25.9)	7.7 (7.1–7.6)	69.3
Centre-North	67,098	27,070	59.7	24.7 (24.5–25.1)	9.0 (8.8–9.1)	63.4
Centre-West	80,577	37,672	53.2	30.0 (29.9–30.1)	12.7 (12.5–12.8)	57.5
Centre-South	34,632	13,550	60.9	25.1 (25.0-25.91)	8.8 ( 8.6-9.0)	64.7
East	79,147	34,560	56.3	26.8 (26.7–26.9)	10.3 (10.2–10.9)	61.6
Hauts-Bassins	109,627	37,969	65.3	30.3 (30.1–30.5)	9.3 (9.3–9.3)	69.5
North	57,273	22,288	61.1	22.6 (22.3–22.8)	8.1 (8.1–8.2)	64.2
Plateau Central	29,433	12,547	57.4	19.7 (19.3–20.0)	7.5 (7.3-8.0)	61.8
Sahel	66,990	30,598	54.3	29.8 (29.7–29.9)	12.2 (12.0-12.5)	59.0
South-West	52,095	32,162	38.3	36.9 (36.3–37.1)	20.6 (20.2–21.4)	44.4
Overall	704,202	290,189	58.8	23.9 (23.1–24.5)	8.9 (8.3-9.0)	63.1

**Table 6:** Posterior estimates of the effect of changes in interventions and climatic factors on parasitaemia risk decline from 2014 to 2017/2018 in Burkina Faso

Predictor	OR (95% BCI)
Difference in proportion of households with at least one ITN for every two people (ITN ownership)	0.98 (0.80–1.16)
Difference in proportion of children who slept under an ITN (ITN use)	0.76 (0.62–0.92)^a^
Difference in ACT coverage	1.09 (0.90–1.31)
Difference in distance to permanent water body	0.87 (0.71–1.04)
Difference in night lights	0.85 (0.57–0.96)^a^
Difference in LST night	0.98 (0.78–1.19)
**Spatial parameters**	
Spatial variance	0.71 (0.35–1.28)
Non-spatial variance	0.44 (0.25–0.78)
Range (km)	270 (130–372)

^a^ Statistically important, estimates are posteriors median, ITN, insecticide-treated net; ACT, artemisinin-based combination therapy; LST, land surface temperature

## Discussion

This study assessed the effects of malaria control interventions and climatic factors simultaneously on the geographical distribution and on the change of malaria parasitaemia risk in Burkina Faso between 2014 and 2017/2018. ITN use had a statistically important effect on the geographical distribution of parasitaemia risk in 2017/2018 but not in 2014. ITN use was also associated with parasitaemia risk decline from 2014 to 2017/2018. Similar results have been reported before [34, 35]. A study conducted in Africa reported also that bednets were effective, mainly because they prevent *Anopheles* mosquitoes from feeding blood, even in case nets are damaged and also because of their capacity to eliminate the disease-carrying vectors [36]. Bednets act as a physical barrier between mosquitoes and the people sleeping under the nets. The nets are treated to repel mosquitoes or kill them when they land on the nets [37]. Bednets have been found to have a significant effect on malaria transmission [38, 39] in children under 5 years of age in additional research conducted in Africa and Papua New Guinea.

The statistical important effect of a decreased coverage of ITNs use between the two time periods could be explained by the fact that the good use of ITNs protect not only individual protection but also contribute to community level protection by reducing the overall population of mosquitoes in an area. This observation is supported by previous studies in Malawi [40] and Mozambique [41].

There was a variation in the effect of ITN across the country. Previous studies in Burkina Faso [19], in Haiti [42] and Uganda [30] reported varying effect of ITN use. A study has showed that even with bednets usage by the entire population, the reduction of malaria transmission would be constrained to a certain minimum level. This is due to the fact that bednets are typically used during the night [43], and studies have documented instances of early mosquito bites occurring when people are outdoors [42, 44]. Interventions’ effects might be affected by many factors, such as human behaviour, mosquito behaviour, pyrethroid vector resistance or the physical integrity of bednets. It should also be noted that bednets have a variable lifespan in terms of physical condition, residual insecticidal effect and perceived usefulness, depending on the geographical and cultural context [34]. For instance, bednets can be effective for up to 20 washings, but their efficacy can be significantly reduced if certain measures are not taken, such as avoiding the use of bleach or hot water when washing them, and avoiding drying them in direct sunlight [45]. Hence, low compliance of bednets use may reduce its potential impact on malaria transmission [19]. A previous study also found that bednets may not always be effective because children are vulnerable during the early evening and early morning hours when they are not protected by the nets or inside the house [46].

ACT coverage was not associated with the geographical distribution and decline of parasitaemia risk from 2014 to 2017/2018. Similar findings have been reported in Angola, Liberia, Senegal, Mozambique, Rwanda, Tanzania [34], Burkina Faso [19] and Uganda [30]. This non-protective effect of ACT might be explained by the fact that this intervention was measured by the proportion of fever episodes rather than clinical malaria cases treated by ACT. As the aetiology of fever in children under 5 years old is multifactorial, causes other than malaria must be considered, such as respiratory viral infections or bloodstream infection, and hence, the effect of ACT is not clear [47, 48]. Nevertheless, at a regional level, this intervention had a stronger effect on malaria reduction in some regions comparing to the national effect. Other studies in Tanzania [49] and Uganda [17] also revealed an important and negative association of ACTs and malaria prevalence. Similarly, Diboulo et al. (2016), in a study conducted in Burkina Faso, have reported that ACT coverage was not important at national level but was statistically important at regional level. This finding could be explained by the fact that the effectiveness of a given intervention is related to both its coverage and transmission levels [19]. The effect of IRS could not be estimated because the survey of 2017/2018 did not have coverage related information.

Rainfall was not found to be an important predictor for the observed change of parasitaemia risk. This result might be related to the data collection period, which was at the beginning of dry season in 2017/2018 and shortly after the end of the rainy season in 2014. Although Roll Back Malaria (RBM) has recommended that surveys take place during the highest transmission season (rainy season), this is not always feasible because of logistical issues and a scarcity of human resources to reach remote areas [34]. LST night was negatively associated with parasitaemia risk in 2014 and 2017/2018. Similar results have been reported before from Burkina Faso [18, 19] and Kenya [13, 16]. This finding might be explained through the optimum temperature at which the mosquito and the parasite develop. Both *Anopheles* and *Plasmodium* are sensitive to temperature. Because mosquitoes are ectotherms, each life stage is dependent on temperature in the developmental and mortality rates [50]. Agyekum et al. (2021) reported that the longevity and survival rate of *An. gambiae* mosquitoes were higher in the rainy season, which is associated with cooler temperatures (17.5 ± 2.9 days) and higher humidity (84.5% ± 10.5%), than in the dry season which is associated with hotter temperatures and lower humidity (7.3 ± 2.8 days and 57.5% ± 14.9%, respectively) [51]. In effect, *Anopheles* infection rates depend on the duration of the reproduction cycle of the parasites in the mosquito, which in turn is related to the temperature [52]. Increasing temperature decreases the longevity of mosquitoes and increases mosquito mortalities, and hence, the transmission decreases. The relationship between temperature and longevity could be explained in two ways. First, higher temperatures may decrease the longevity by speeding the reaction rate of various metabolic processes that affect development and life history. Second, higher temperatures might heighten the damage caused by the by-products of metabolism, such as reactive oxygen species [51]. It has also been reported that *P. falciparum* only develop between 15 and 35 °C, the optimum being at 25 °C. The more one deviates from this optimum, the longer the duration of the parasite’s reproduction cycle, and hence, the infection rate of *Anopheles* decreases [52]. Additional studies have demonstrated that malaria parasites have a temperature-dependent development curve that does not match up with the mosquito temperature curve. This suggest, for example, that if the optimal temperature for parasite development is higher than the range preferred by mosquitoes, this could result in slower development of the parasite in the mosquito, and hence, reducing the malaria transmission. In contrary, if the mosquito’s preferred temperature is higher than the optimal range for parasite development, the mosquito may survive longer, leading to an increase in the transmission rate [50]. However, other studies have reported that an increase of LST night was associated with an increase of parasitaemia risk in Uganda and Cameroon [15, 53].

An increase of night light, which is a proxy for urbanization [54], was associated with a reduction of malaria parasite prevalence. This result is consistent with previous studies in Africa [15, 19, 55] that have shown that children living in urban areas are at a lower risk of malaria compared to children residing in rural areas where *An. gambiae* is the predominate vector. Urbanization can change malaria transmission patterns over longer time scales by creating more or less favourable microclimatic conditions and habitats for malaria vectors, depending on the species [56, 57]. For example, urban development can eliminate mosquito habitats and reduce malaria incidence. In some cases, increases have been observed in peri-urban malaria as a result of economic migration and the creation of novel breeding habitats [58]. Urbanization affects anopheline species diversity, mosquito density, survival rates and infection rates with *P. falciparum* and the frequency with which mosquitoes bite people are all affected. Hence, fewer people acquire malaria infection, become ill or die of its consequences in urban areas [59]. Furthermore, urban areas have stronger networks of better equipped higher level health facilities both public and private, while rural area often lack medical facilities in term of quantity and quality.

At higher altitudes, malaria transmission decreased. Similarly, a study in Tanzania has shown that the primary effect of increasing altitude was a log-linear reduction in vector abundance and, to a lesser extent, a reduction in the proportion of infective mosquitoes [60]. In the lowland regions of sub-Saharan countries, malaria is holoendemic with perennial transmission. On the contrary, highland areas are known to be malaria hypoendemic, due to climate (low temperature and relative humidity), which is not suitable for anopheline development and their reproductive fitness [61]. At higher elevations, temperatures have also historically been too cold for *Plasmodium* to be transmitted [62]. Therefore, the risk of malaria parasite decreases with altitude due to an impact of temperature on the cycle of the *Anopheles* mosquito of the parasite. This risk is not linear due to the many other determinants that influence the epidemiology of malaria at a given altitude [63].

Our results showed a strong reduction in the number of under-5-year-old children infected with *Plasmodium* in Burkina Faso when comparing MIS 2014 with 2017/2018, which may underline the effect of ITN use and changing climatic conditions. The highest posterior probability of reduction in parasitaemia risk occurred in the region of Centre and the lowest in the region of South-West. In effect, the Centre is the capital and the most urbanized setting in Burkina Faso where the effect of ITN use was important. In contrast, the region of South-West is less urbanized with high endemicity and lower average temperature [64]. South-West has also a longer rainy season and is characterised by many swamps, which act as reservoirs for malaria parasites [65]. Finally, the decrease of spatial variance associated to an increase of the spatial range from 2014 to 2017/2018 can be explained by the fact that more climatic factors were linked to malaria parasite prevalence in 2014 and therefore the residual spatial structure in the model was lower compared to 2017/2018.

A main limitation in this study is that the MIS of 2014 and of 2017/2018 were carried out during the early dry and dry season, respectively. Therefore, it is likely that the difference in transmission seasons, overestimates the reduction in malaria parasitaemia prevalence.

## Conclusion

Parasitaemia prevalence was considerably lower in 2014 compared to 2017/2018. During the same time the coverage of bednets interventions decreased and that of ACTs increased. Annual mean temperature remained the same but rainfall was lower mainly as the second survey was carried out in the dry season. Temperature had a statistically important negative association with the geographical distribution of parasitaemia prevalence in both surveys, however the effects of malaria interventions (i.e. proportion of children who slept under an ITN) were negative and statistically important only in 2017/2018. The latter was also related with the geographical distribution of the changes in malaria prevalence after adjusting for changes in temperature. The decline of the parasite prevalence distribution was related to the ACT coverage at regional but not national level.

The malaria control programme should pay attention to the potential hotspot regions of South-West where the posterior probability of reduction in parasitaemia risk was lower than 20%. The effectiveness of all interventions were lower than that of national average or not statistically important in South-West and Boucle du Mouhoun. To accurately evaluate the relative effects of climate and malaria interventions to malaria parasite declines, MIS surveys should be carried out at the same time of the year. In addition, longer time series analysis can help to more fully understand the differential effects of a changing climate on malaria parasite risk, to develop forecasting models, and to improve the ability to anticipate and also mitigate the future effects of climate change on malaria outbreaks.

### Electronic supplementary material

Below is the link to the electronic supplementary material.

Supplementary Material 1

Supplementary Material 2

Supplementary Material 3

## Data Availability

The study data are available upon request from the Demographic and Health Surveys programmes (https://dhsprogram.com/).
