# Supplementary material for: Relative effects of climate factors and malaria control interventions on changes of parasitaemia risk in Burkina Faso from 2014 to 2017/2018
Source: BMC Infect Dis. 2024 Feb 7;24:166. doi: 10.1186/s12879-024-08981-2 (PMC10848559; doi:10.1186/s12879-024-08981-2)
Supplement: Supplementary file 1 — Supplementary Material 1 [file 12879_2024_8981_MOESM1_ESM.docx]

**Table 3A: Malaria interventions coverage by region in 2014 and 2017/2018 in percentage (%)**

| Interventions/ Periods | | Prevalence | Proportion of households with at least one | Proportion of households with at least one ITN for every two people | Proportion of population with access to an ITN in their household | Proportion of the population that slept under an ITN the previous night | Proportion of children under five years old who slept under an ITN the previous night | Proportion of pregnant women who slept under an ITN the previous night | Proportion of existing ITNs used the previous night | Proportion of households sprayed in the last 6 months | Proportion of children with fever treated with ACT | Proportion of households with at least one ITN and/or sprayed by IRS in the last 12 months |
| --- | --- | --- | --- | --- | --- | --- | --- | --- | --- | --- | --- | --- |
| Boucle du Mouhoun | 2014 | 55.44 | 89 .60 | 40 .49 | 71 .60 | 68 .25 | 77 .23 | 76 .85 | 95 .82 | 0 .20 | 18 .75 | 89 .61 |
|  | 2017/2018 | 17.55 | 82 .20 | 35 .45 | 64 .40 | 49 .30 | 59 .20 | 76 .10 | 73 .50 | 0 .00 | 28 .40 | 82 .20 |
| Cascades | 2014 | 49.3 | 88 .10 | 44 .60 | 72 .90 | 68 .30 | 77 .30 | 89 .40 | 90 .10 | 0 .60 | 39 .60 | 88 .10 |
|  | 2017/2018 | 14.26 | 83 .40 | 31 .30 | 64 .30 | 59 .50 | 72 .30 | 76 .30 | 83 .90 | 0 .00 | 46 .00 | 83 .00 |
| Centre | 2014 | 9.30 | 87 .10 | 53 .50 | 74 .30 | 61 .10 | 68 .30 | 68 .50 | 78 .40 | 0 .60 | 13 .50 | 87 .10 |
|  | 2017/2018 | 6.60 | 81 .70 | 46 .60 | 70 .20 | 58 .60 | 64 .80 | 83 .30 | 84 .30 | 0 .00 | 35 .30 | 81 .70 |
| Centre-East | 2014 | 39.81 | 95 .70 | 50 .50 | 81 .20 | 79 .50 | 87 .30 | 87 .00 | 88 .90 | 0 .00 | 11 .30 | 95 .70 |
|  | 2017/2018 | 12.09 | 63 .20 | 26 .60 | 48 .70 | 39 .80 | 48 .90 | 62 .30 | 81 .90 | 0 .00 | 41 .20 | 63 .20 |
| Centre-West | 2014 | 57.86 | 90 .90 | 44 .90 | 75 .60 | 62 .20 | 73 .70 | 63 .70 | 77 .60 | 0 .00 | 10 .10 | 90 .90 |
|  | 2017/2018 | 23.28 | 83 .30 | 40 .40 | 68 .10 | 43 .80 | 58 .70 | 46 .10 | 60 .70 | 0 .00 | 60 .60 | 83 .30 |
| Centre-North | 2014 | 41.46 | 95 .60 | 45 .60 | 78 .80 | 73 .10 | 82 .90 | 86 .50 | 90 .80 | 0 .00 | 6 .10 | 95 .60 |
|  | 2017/2018 | 17.32 | 79 .20 | 31 .10 | 61 .40 | 47 .40 | 57 .70 | 44 .50 | 88 .00 | 0 .00 | 50 .80 | 79 .20 |
| Centre-South | 2014 | 41.71 | 89 .50 | 59 .40 | 80 .20 | 74 .80 | 81 .90 | 81 .70 | 86 .30 | 0 .00 | 12 .70 | 89 .50 |
|  | 2017/2018 | 11.5 | 70 .80 | 29 .30 | 55 .60 | 44 .30 | 56 .10 | 48 .40 | 81 .00 | 0 .00 | 27 .30 | 70 .80 |
| East | 2014 | 54.08 | 78 .20 | 43 .30 | 65 .70 | 63 .90 | 74 .30 | 82 .90 | 92 .80 | 0 .00 | 5 .80 | 78 .20 |
|  | 2017/2018 | 16.36 | 69 .00 | 25 .10 | 50 .90 | 41 .10 | 53 .80 | 56 .40 | 84 .20 | 0 .00 | 43 .40 | 69 .00 |
| Haut-Bassins | 2014 | 40.92 | 93 .70 | 61 .30 | 84 .10 | 73 .80 | 80 .10 | 84 .00 | 82 .30 | 0 .40 | 19 .60 | 93 .70 |
|  | 2017/2018 | 11.04 | 71 .10 | 26 .20 | 52 .90 | 40 .80 | 49 .90 | 48 .90 | 80 .20 | 0 .00 | 28 .50 | 71 .10 |
| North | 2014 | 42.48 | 95 .50 | 48 .70 | 80 .30 | 65 .50 | 75 .80 | 79 .70 | 77 .00 | 1 .00 | 17 .80 | 95 .50 |
|  | 2017/2018 | 34.00 | 79 .90 | 28 .90 | 60 .00 | 50 .80 | 65 .40 | 55 .60 | 78 .50 | 0 .00 | 44 .20 | 79 .90 |
| Plateau-Central | 2014 | 33.66 | 92 .40 | 46 .60 | 77 .70 | 64 .60 | 65 .80 | 84 .80 | 88 .40 | 0 .20 | 6 .10 | 92 .40 |
|  | 2017/2018 | 8.56 | 77 .90 | 35 .00 | 60 .60 | 40 .20 | 47 .30 | 61 .50 | 68 .60 | 0 .00 | 53 .50 | 77 .90 |
| Sahel | 2014 | 54.10 | 85 .60 | 33 .60 | 65 .70 | 61 .20 | 70 .50 | 55 .80 | 86 .40 | 0 .00 | 10 .30 | 85 .60 |
|  | 2017/2018 | 23.41 | 75 .50 | 40 .30 | 62 .30 | 33 .80 | 41 .90 | 18 .70 | 54 .20 | 0 .00 | 37 .90 | 75 .50 |
| South-West | 2014 | 60.22 | 84 .80 | 47 .00 | 71 .50 | 59 .60 | 66 .00 | 74 .20 | 82 .60 | 3 .50 | 24 .70 | 85 .10 |
|  | 2017/2018 | 39.00 | 59 .00 | 23 .40 | 42 .90 | 27 .20 | 31 .90 | 38 .70 | 73 .70 | 0 .00 | 61 .50 | 59 .00 |

**Table 3B: Posterior odds ratios (ORs) and 95% Bayesian credible intervals (BCIs) estimated by multivariate geostatistical logistic regression models with interactions terms fitted to MIS 2014 and 2017/2018 data from Burkina Faso**

| **Predictor** | **MIS 2014**  **OR (95% BCI)** | **MIS 2017/2018**  **OR (95% BCI)** |
| --- | --- | --- |
| LST night | 0.72 (0.64-0.93)^a^ | 0.89 (0.81-0.96)^a^ |
| Night light | 0.47 (0.38-0.59)^a^ | 0.49 (0.37-0.64)^a^ |
| Distance to permanent water body | 0.83 (0.72-0.94)^a^ | 0.71 (0.57-0.85)^a^ |
| Altitude**^*^**  [201-307 m]  [307-545 m] | 1.00  0.72 (0.51-0.96)^a^ | - |
| Proportion of households with at least one ITN for every two people (ITN ownership) | 1.06 (0.24-1.48) | 1.04 (0.86-1.20) |
| Proportion of children who slept under ITN (ITN use) | - | 0.82 (0.70- 0.96)^a^ |
| ACTs | - | 1.22 (0.93-1.40) |
| **Interactions** |  |  |
| ACTs x LST night | 1.11 (0.91-1.33)) | 1.04 (0.90-1.29) |
| ITN ownership x LST night | 0.93 (0.78-1.10) | 0.96 (0.78-1.14) |
| ITN use x LST night | 0.99 (0.80-1.20) | 1.04 (0.85-1.27) |
| **Spatial parameters** |  |  |
| Spatial variance | 0.41 (0.19-0.71) | 0.32 (0.15-0.61) |
| Non spatial variance | 0.31 (0.16- 0.49) | 0.29 (0.14-0.48) |
| Range (km) | 129.4 (57.1-367.5) | 303.7 (189.2-371.1) |

^a^Statistically important effect, ^*^ The cutoff is based on the tertiles of the distribution of altitude at the surveys locations, range is the minimum distance at which the spatial correlation in no longer or less than 5%, ITN: insecticide-treated net, ACT: artemisinin-based combination therapy, LST: land surface temperature
