# Supplementary material for: Relative effects of climate factors and malaria control interventions on changes of parasitaemia risk in Burkina Faso from 2014 to 2017/2018
Source: BMC Infect Dis. 2024 Feb 7;24:166. doi: 10.1186/s12879-024-08981-2 (PMC10848559; doi:10.1186/s12879-024-08981-2)
Supplement: Supplementary file 2 — Supplementary Material 2 [file 12879_2024_8981_MOESM2_ESM.docx]

**Detailed descriptions of the analysis**

**A. Bivariate analysis**

A bivariate geostatistical model for each time point was developed to assess the association between the prevalence and each one of the predictors (climatic factors and interventions). Let $Y(s_{i})$ be the number of children below the age than 5 years who tested positive in cluster $s_{i}$ in the survey, and $N\left( s_{i} \right),$ the total number of children tested. $Y(s_{i})$ follows a Binomial distribution, that is, $Y\left( s_{i} \right)|N\left( s_{i} \right),\pi(s_{i})\sim Bin(N\left( s_{i} \right),\pi\left( s_{i} \right))$ $\forall$i ∈ *1,…,n_1_*, where ***s****=*{*s_1_, s_2_,…,s_n_*} is the set of locations surveyed, $s_{i}\subset R^{2}$ and $\pi(.)$ indicates the parasitaemia risk. The bivariate model is formulated as following:

$\mathrm{logit}\left( \pi\left( s_{i} \right) \right)=\beta^{T}X\left( s_{i} \right)+\omega\left( s_{i} \right)+v(s_{i})$, where $X$ is the predictor, $\beta$ is the coefficient parameter, $\omega$the spatial random effect and $v$ represents the non-spatial random effect.

**B. Estimating parasitaemia risk at two survey time points (Multivariate analysis)**

A multivariate geostatistical model was then developed to assess the effect of environmental/climatic factors and interventions using the significant variables from the bivariate analysis, on the geographical distribution of parasitaemia risk for the first survey. Let $Y_{1}(s_{i})$ be the number of children below the age than 5 years who tested positive in cluster $s_{i}$ in the first survey, and $N_{1}\left( s_{i} \right),$ the total number of children tested. We assume that $Y_{1}(s_{i})$ follows a Binomial distribution, that is, $Y_{1}\left( s_{i} \right)|N_{1}\left( s_{i} \right),\pi_{1}(s_{i})\sim Bin(N_{1}\left( s_{i} \right),\pi_{1}\left( s_{i} \right))$ $\forall$i ∈ *1,…,n_1_*, where ***s****=*{*s_1_, s_2_,…,s_n_*} is the set of locations surveyed, $s_{i}\subset R^{2}$ and $\pi_{1}(.)$ indicates the parasitaemia risk. A Bayesian geostatistical model to analyze parasitaemia risk was formulated as follows:

$\mathrm{logit}\left( \pi_{1}\left( s_{i} \right) \right)=\beta_{1}^{T}X_{1}\left( s_{i} \right)+\omega_{1}\left( s_{i} \right)+v_{1}(s_{i})$, where $X_{1}\left( s_{i} \right)$ is the set of climatic and interventions variables at location$s_{i}$, $\boldsymbol{\beta}_{\boldsymbol{1}}$ *= (*$\beta_{11}, \beta_{12},\ldots,\beta_{1k}$*)^T^* is the vector of regression coefficients and ${\boldsymbol{\omega}_{\boldsymbol{1}}=(\omega_{1}\left( s_{1} \right), \omega_{1}\left( s_{2} \right),\ldots, \omega_{1}(s_{n1}))}^{T}$ is a zero-mean latent spatial process that follows a multivariate normal distribution, that is, $\boldsymbol{\omega}_{\boldsymbol{1}}\sim MVN(0,{\sigma_{1}^{2}R}_{1})$. ${\boldsymbol{v}_{\boldsymbol{1}}=(v_{1}\left( s_{1} \right), v_{1}\left( s_{2} \right),\ldots, v_{1}(s_{n1}))}^{T}$ is the non-spatial random effect which follows a normal distribution, that is $\boldsymbol{v}_{\boldsymbol{1}}\sim N(0,{\sigma_{1}^{2}})$.R_1_ is the correlation matrix defined by an exponential parametric function of the distance $d_{\mathrm{ij}}$ between two location $s_{i}$ and$s_{j}$ that is, $R(s_{i}, s_{j})=exp(-d_{\mathrm{ij}}\rho_{1})$. The parameter $\sigma_{1}^{2}$ is the spatial variation and $\rho_{1}$ is a smoothing parameter that controls the rate of correlation decay with increasing distance. The range parameter was calculated by the ratio $\frac{3}{\rho_{1}}$ to estimate the minimum distance beyond which spatial correlation is negligible (<5%). Following standard formulation of Bayesian regression models, we assumed vague priors; an inverse-gamma for $\sigma_{1}^{2}$, a gamma prior distributions for $\rho_{1}$, and non-informative Gaussian distributions with mean 0 and variance 100 for the regression coefficients.

Thus, $\sigma_{1}^{2}$~IG (0.01, 0.01), $\rho_{1}$~Gamma (2.01, 1.01), $\beta_{1k}$~N(0, 10^2^), *k=1,…,K*

To produce a smooth map, Bayesian kriging was employed to predict parasitaemia risk at unsampled locations on a 2 x 2 km^2^ grid using the predictive posterior distribution, p(Y_0_|Y) = $\int p\left( Y_{0} | \beta_{1}, \boldsymbol{\omega}_{\boldsymbol{0}} \right)p\left( \boldsymbol{\omega}_{\boldsymbol{0}} | \boldsymbol{\omega}_{\boldsymbol{1}},\sigma_{1}^{2}, \rho_{1} \right)p\left( \beta_{1}, \boldsymbol{\omega}_{\boldsymbol{1}},\rho_{1}, \sigma_{1}^{2} | Y_{1}\left( s_{i} \right) \right)d\boldsymbol{\beta}_{\boldsymbol{1}}d\boldsymbol{\omega}_{\boldsymbol{0}}d\boldsymbol{\omega}_{\boldsymbol{1}}d\sigma_{1}^{2}d\rho_{1}$, where

***Y_0_***= ($Y_{1}\left( s_{01} \right),Y_{1}\left( s_{02} \right),\ldots,Y_{1}\left( s_{0l} \right)$)^T^ is the number of infected children at unsampled location $s_{0}$ *= {*$s_{01}, s_{02}, \ldots,s_{0l}\}$ *,*

$\boldsymbol{\omega}_{\boldsymbol{0}}$ is the spatial random effect at $s_{0}$*.* The distribution of $\omega_{0}$ given $\omega_{1}$ is multivariate normal, that is, $p\left( \boldsymbol{\omega}_{\boldsymbol{0}} | \boldsymbol{\omega}_{\boldsymbol{1}},\sigma_{1}^{2}, \rho_{1} \right)$=MVN ($R_{01}R_{11}^{-1}U, {\sigma_{1}^{2}(R}_{01}-R_{01}R_{11}^{-1}R_{10})$), with$R_{11}$= cor($\boldsymbol{\omega}_{\boldsymbol{1}},\boldsymbol{\omega}_{\boldsymbol{1}}$), $R_{01}$=$R_{10}^{T}$= cor($\boldsymbol{\omega}_{\boldsymbol{0}},\boldsymbol{\omega}$) and p($Y\left( s_{0i} \right)|\beta_{1},\omega\left( s_{0i} \right)$) ~$Bin(Y\left( s_{0i} \right),\pi_{0}\left( s_{0i} \right))$, and thus logit ($\pi_{0}\left( s_{0i} \right))$=${\beta_{1}}^{T}X\left( s_{0i} \right) +\omega\left( s_{0i} \right)$.

For mapping purposes, predictions were made for 70,224 pixels covering a regular grid at 2x2 km^2^ spatial resolution of Burkina Faso.

Using a geostatistical model similar to the one described above, estimates of malaria risk were obtained for the second survey. Similarly, a Binomial distribution was assumed for the number of positive children$Y_{2}\left( s_{i}^{'} \right)$, that is$Y_{2}\left( s_{i}^{'} \right)|N_{2}\left( s_{i}^{'} \right),\pi_{2}\left( s_{i}^{'} \right)\sim Bin\left( N_{2}\left( s_{i}^{'} \right),\pi_{2}\left( s_{i}^{'} \right) \right),\forall i \in1,\ldots,n_{2},$

where $\mathbf{s}^{\mathbf{'}}=\{s_{1}^{'}, s_{2}^{'},\ldots,s_{n_{2}}^{'}\}$ is the set of locations sampled in the second survey, which is different from$\mathbf{s}$. $\pi_{2}(s_{i}^{'})$ was modelled as a function of the climatic factors and a spatial process $\boldsymbol{\omega}_{\boldsymbol{2}}$, that is, $\boldsymbol{\omega}_{\boldsymbol{2}}\sim MVN(0,{\sigma_{2}^{2}R}_{2})$ with spatial variance $\sigma_{2}^{2}$ and scaling parameter $\rho_{2}$. On the logit scale, this takes the form,$\mathrm{logit}\left( \pi_{2}\left( s_{i}^{'} \right) \right)=\beta_{2}^{T}X_{2}\left( s_{i}^{'} \right)+\omega_{2}\left( s_{i}^{'} \right)+v_{1}(s_{i}^{'})$. Also, prediction of parasitaemia risk for the second survey was carried out using the 2x2 km^2^ resolution grid described above.

**C. Modeling the effects of interventions and climatic factors on the change of parasitaemia risk**

The change of parasitaemia risk was modelled on the logit scale as a function of the difference in climatic/environmental conditions between the two survey times, the effect of intervention coverage, in the second survey, that is:

$\mathrm{logit}\left( \pi_{2}\left( s_{i}^{'} \right) \right)=Z\left( s_{i}^{'} \right)+{{\beta(X}_{2}\left( s_{i}^{'} \right)-X_{1}\left( s_{i}^{'} \right))}^{T}+\alpha_{1}\mathrm{ITNownership}\left( s_{i}^{'} \right)+\alpha_{2}\mathrm{ITNuse}\left( s_{i}^{'} \right)+\alpha_{3}\mathrm{ACT}\left( s_{i}^{'} \right)+\omega_{c}\left( s_{i}^{'} \right)+v_{c}(s_{i}^{'})$, where Z($s_{i}^{'}$) = logit($\pi_{1}({s_{i}}^{'})$), $ITN ownership\left( s_{i}^{'} \right)$,$ITN use\left( s_{i}^{'} \right)$ is the coverage of ITN identified through a variable selection procedure, $ACT(s_{i}^{'})$ is the proportion of fevers treated with any ACT, and $\omega_{c}(s_{i}^{'})$ corresponds to the latent spatial process, that is, $\omega_{c}\sim MVN(0,{\sigma_{c}^{2}R}_{c})$ with spatial variance $\sigma_{c}^{2}$. $v_{c}(s_{i}^{'})$ is the non-spatial random effect, which is $v_{c}\sim N(0,{\sigma_{c}^{2}})$.The coefficients $\alpha_{1}$*,* $\alpha_{2}$ and $\alpha_{3}$ measure the effect of interventions on the change in parasitaemia risk, thus, $exp(\alpha_{1})$, $exp(\alpha_{2})$ and $exp(\alpha_{3})$ are the expected change in odds of parasitaemia (second survey versus first survey) associated with a 1% increase in the coverage of ITNs and ACT, respectively.

We assume an inverse gamma prior distribution for $\sigma_{c}^{2},$ a gamma distribution for the parameter $\rho_{c}$, and normal priors for the regression coefficients $\beta$*,* $\alpha_{1}$*,* $\alpha_{2}$*,* $\alpha_{3}$.

Parasitaemia risk during the first survey $\pi_{1}(.)$ was not directly available at locations $s^{'}$of the second survey. We addressed this spatial misalignment problem by predicting parasitaemia risk during the first period at the locations of the second survey using the Bayesian kriging.

**D. Estimating the probability of reduction in parasitaemia risk**

Conditional on the data and the model parameters, the predictive density for each time point can be expressed as:

$$P\left( Y_{t}^{0} | Y_{t},N_{t} \right)=\int P\left( Y_{t}^{0} | \beta_{t},\omega_{t}^{0} \right)P\left( \omega_{t}^{0} | \omega_{t},\sigma_{t}^{2},k_{t} \right)P\left( \beta_{t},\omega_{t},\sigma_{t}^{2},k_{t} | Y_{t},N_{t} \right)d\beta_{t}d\omega_{t}^{0}d\omega_{t}d\sigma_{t}^{2}dk_{t},$$

where $Y_{t}^{0}=\left( Y_{t}\left( s_{1}^{0} \right),Y_{t}\left( s_{2}^{0} \right),\ldots,Y_{t}\left( s_{m}^{0} \right) \right)$ are the predicted number of positives in each pixel $s_{i}^{0} \forall i \epsilon1,\ldots,m$ and time point *t*, *t* = 1*,*2, and $P(\beta_{t},\omega_{t},\sigma_{t}^{2},k_{t}$| $Y_{t}, N_{t})$ is the joint posterior distribution of parameters and hyperparameters while

$\omega_{t}=(\omega_{t}\left( s_{1}^{0} \right),\omega_{t}\left( s_{2}^{0} \right),\ldots,\omega_{t}\left( s_{m}^{0} \right))$ is the vector of the spatial process at new sites.

Conditional on the spatial process and regression parameters, $Y_{t}\left( s_{i}^{0} \right)\sim Bin\left( N_{t}\left( s_{i}^{0} \right),\pi_{t}\left( s_{i}^{0} \right) \right),$ with risk $\pi_{t}(s_{i}^{0})$ $s_{i}^{0})$, given by $logit\left( \pi_{t}\left( s_{i}^{0} \right) \right)=\beta_{t}X_{t}\left( s_{i}^{0} \right)+\omega_{t}(s_{i}^{0})$ and *N_t_* ( $s_{i}^{0})$ indicates the population of children living in the pixel $s_{i}^{0}$*_._* To estimate the probability of risk reduction at each pixel we have compared $\pi_{1}\left( s_{i}^{0} \right)$ with $\pi_{2}\left( s_{i}^{0} \right), \forall i\epsilon1,\ldots,m$ and calculated$P\left( \pi_{2}\left( s_{i}^{0} \right)<\pi_{1}\left( s_{i}^{0} \right) \right)$. Furthermore, we have estimated the total number of children infected during the first and second survey period $(\sum_{i=1}^{m} Y_{1}\left( s_{i}^{0} \right) and \sum_{i=1}^{m} Y_{2}\left( s_{i}^{0} \right)$respectively). We made use of population data provided by WorldPop that consist of spatial estimations of number of children less than 5 years of age per 1km^2^.

$$P\left( Y_{t}^{0} | Y_{t},N_{t} \right)=\int P\left( Y_{t}^{0} | \beta_{t},\omega_{t}^{0} \right)P\left( \omega_{t}^{0} | \omega_{t},\sigma_{t}^{2},k_{t} \right)P\left( \beta_{t},\omega_{t},\sigma_{t}^{2},k_{t} | Y_{t},N_{t} \right)d\beta_{t}d\omega_{t}^{0}d\omega_{t}d\sigma_{t}^{2}dk_{t},$$

**E. Spatially varying interventions effects**

In order to estimate the intervention effects at regional level and account for potential interactions with endemicity levels, a second model was fitted in which we estimated intervention effects at regional level. The model was expressed as;

$\mathrm{logit}\left( \pi_{2}\left( s_{i}^{'} \right) \right)=Z\left( s_{i}^{'} \right)+\beta\left( X_{2}\left( s_{i}^{'} \right)-X_{1}\left( s_{i}^{'} \right) \right)+\alpha_{1}\left( A_{s_{i}^{'}} \right)\mathrm{ITNownership}\left( s_{i}^{'} \right)+\alpha_{2}\left( A_{s_{i}^{'}} \right)\mathrm{ITNuse}\left( s_{i}^{'} \right)+\alpha_{3}\left( A_{s_{i}^{'}} \right)\mathrm{ACT}\left( s_{i}^{'} \right)+\omega_{c}\left( s_{i}^{'} \right)+v_{c}(s_{i}^{'})$.

The effects of interventions are defined at regional level and denoted as $\alpha_{k}\left( A_{s_{i}^{'}} \right), (k=1,2,3)$ where $A_{s_{i}^{'}}$ is the region where $s_{i}^{'}$ falls. Each $\alpha_{k}(A_{i})$ was written as the sum of a conditional autoregressive effect that takes into account the similarity of the effects across the regions and an independent random component,

That is$\alpha_{k}\left( A_{i} \right)= \alpha_{k}^{c}\left( A_{i} \right)+\varepsilon_{k}(A_{i})$, where $p(\alpha_{k}^{c}\left( A_{i} \right)|\alpha_{k}^{c}\left( A_{j} \right), i\neq j, \tau_{k})\equiv N(\frac{1}{n_{i}}\sum_{i\sim j} \alpha_{k}^{c}\left( A_{j} \right),\frac{\sigma_{k}^{2}}{n_{i}})$ with $i\sim j$ indicates the $A_{j}$ areas neighboring $A_{i}$ and $\varepsilon_{k}\left( A_{i} \right)\sim N(0,\sigma_{\varepsilon}^{2})$.
