# Supplementary material for: Relative effects of climate factors and malaria control interventions on changes of parasitaemia risk in Burkina Faso from 2014 to 2017/2018
Source: BMC Infect Dis. 2024 Feb 7;24:166. doi: 10.1186/s12879-024-08981-2 (PMC10848559; doi:10.1186/s12879-024-08981-2)
Supplement: Supplementary file 3 — Supplementary Material 3 [file 12879_2024_8981_MOESM3_ESM.docx]

**Malaria prevalence and interventions coverage by regions in 2014 and 2017/2018**





***2014***

***2014***

***2017/2018***

***2017/2018***

***2014***

***2017/2018***

***2017/2018***

***2014***

***2014***

***2017/2018***

**Figure 1A**: Proportion of households with at least one ITN for every two people (A&B), proportion of children under 5 years old who slept under an ITN (C&D), Proportion of fever episodes treated with ACT (E&F), Proportion of existing ITNs used the previous night (G&H), Prevalence (I&J)

**Monthly average climatic factors (rainfall, day land surface temperature and night land surface temperature) over the past decade**


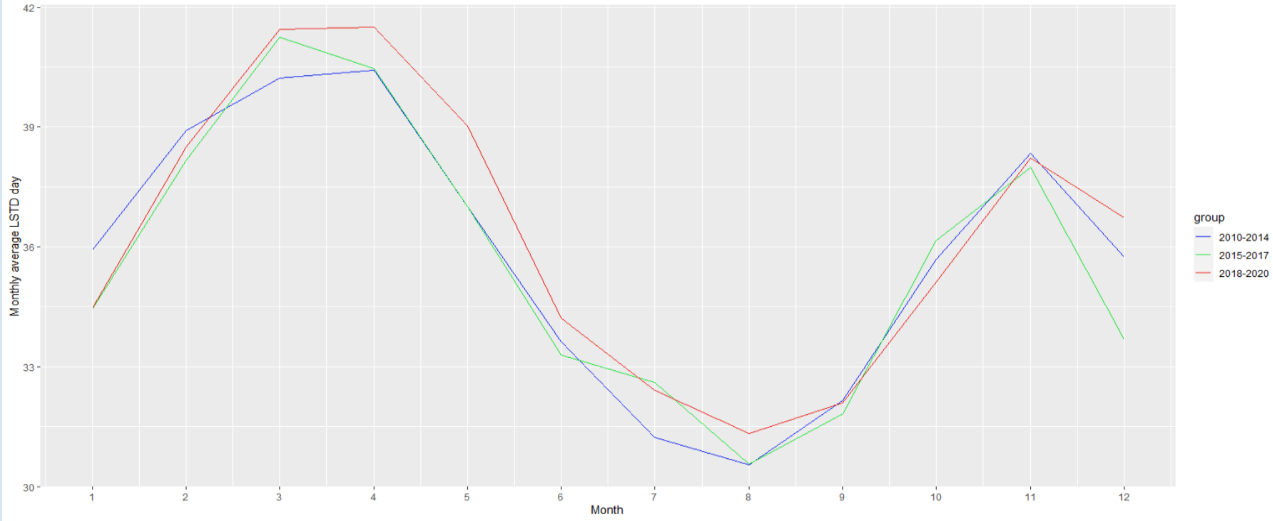


(a)


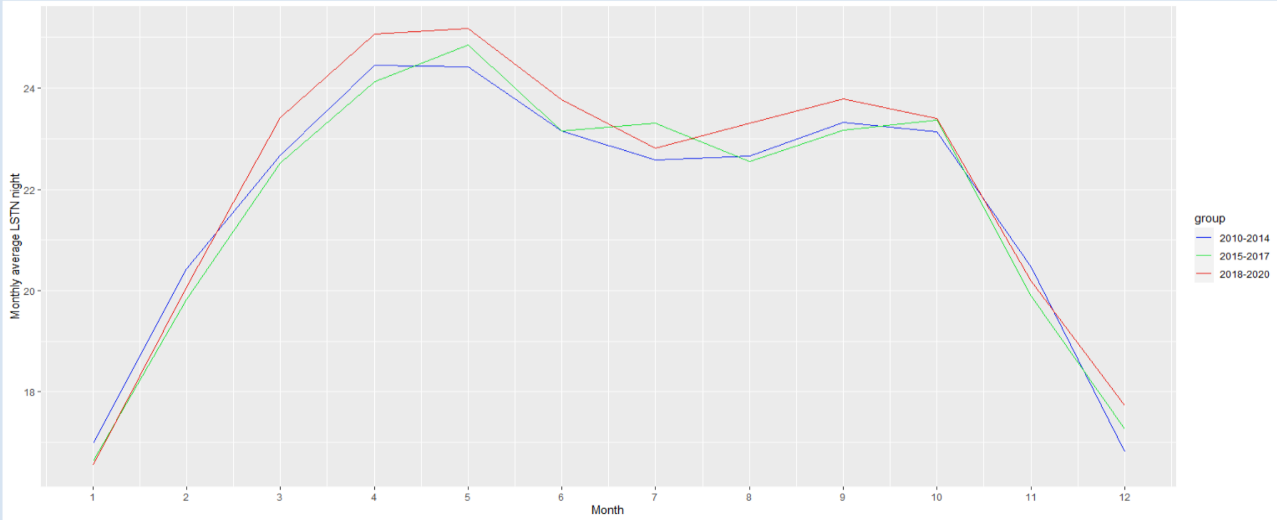


(b)

(c)


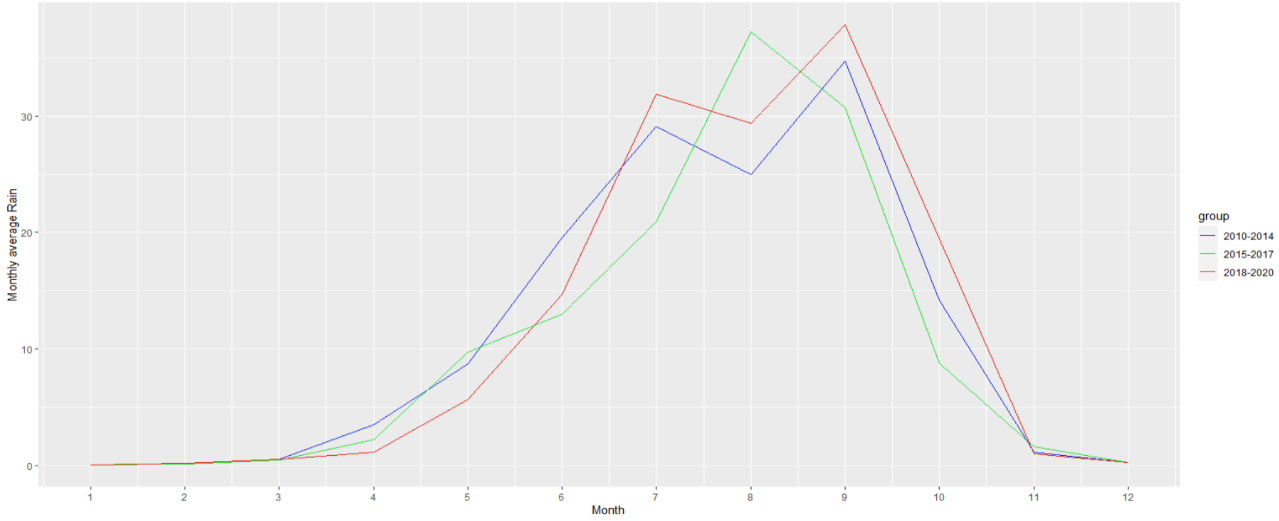


**Figure 1B**: Monthly average of Day LST (a), Night LST (b), Rainfall (c) from 2010 to 2020.
